# 40-years of relative age effects: life is not fair!

**DOI:** 10.3389/fspor.2024.1516173

**Published:** 2025-02-04

**Authors:** Roger H. Barnsley

**Affiliations:** Thompson Rivers University, Kamloops, BC, Canada

**Keywords:** relative age effect, RAE, hockey, soccer, replication, mitigation

## Abstract

This paper explores the Relative Age Effect (RAE) after nearly 40 years since its initial examination in sports. Two original studies identified significant participation differences between relatively older and younger players in age-grouped elite hockey and soccer. In the current study, we replicate the original analyses using 2023 data. By comparing data from the original studies and 2023, focusing on Major Junior A hockey in North America and the Under-17 and Under-20 World Soccer Tournaments, we observe remarkably similar RAE patterns. For instance, both the original and the 2023 studies indicate that about 40% of elite young adult players were born in the first quarter of the age cohort, compared to just 10% in the last quarter. This paper underscores the ongoing advantages and disadvantages created by RAE and calls for greater focus on strategies to mitigate its unfair effects in sports and education.

## Introduction

In 2008, Malcolm Gladwell published “Outliers: The Story of Success” which was met with overwhelming interest and worldwide sales of over 1.6 million copies (1). Chapter one of “Outliers” was entitled “The Matthew Effect” and yet, was substantially influenced by the research article “Hockey Success and Birthdate: The Relative Age Effect” (2). This article, which was the first to use the term “Relative Age Effect” (RAE), reported a striking relationship between a hockey player's success, and their relative standing in an age cohort.

In 2022, Malcolm Gladwell contacted me (3). At this time, Gladwell was producing a podcast entitled “Revisionist History”. He explained that he was working on an update to “Outliers” and would again like to discuss RAEs. Our interview took place in January 2022 and began with Gladwell asking me to join him in looking at the player roster from the 2020–21 Canadian national junior hockey team. Together, we read aloud the players' birthdates. As we finished, there were a few moments of silence and then Gladwell said, “Not much has changed, has it?”. This quick peek at the pattern of these players' birthdates seemed to indicate that they were remarkably like the 1983 player rosters (2).

Gladwell and I shared our disappointment that after 40 years, it appeared that RAEs in hockey had continued unabated. If little, or no progress, in mitigating RAEs had been achieved, then it would seem reasonable to assume that parents and sport administrators were either unaware of the impact of RAEs, or how to bring about change. Surely, in the context of this special issue which celebrates the 40th anniversary of the “Relative Age Effect”, it is appropriate, if not essential, to initiate a broader discussion on “Has anything changed?”^1^

## Hockey and soccer: the early studies^2^

In 1984 Simon Grondin reported birthdate effects in hockey and volleyball (5). Unaware of these findings, in that same year Paula Barnsley observed unusual and significant patterns of birthdates in the rosters of Canadian Major Junior A hockey players. Following her observation, Paula and I reviewed National Hockey League (NHL) players' birthdates. The result, based on 715 players from the 1982/83 team rosters, indicated that 61.8% of the players were born in the months of January through June, whereas only 38.2% were born in the months of July–December.

**Figure 1:**
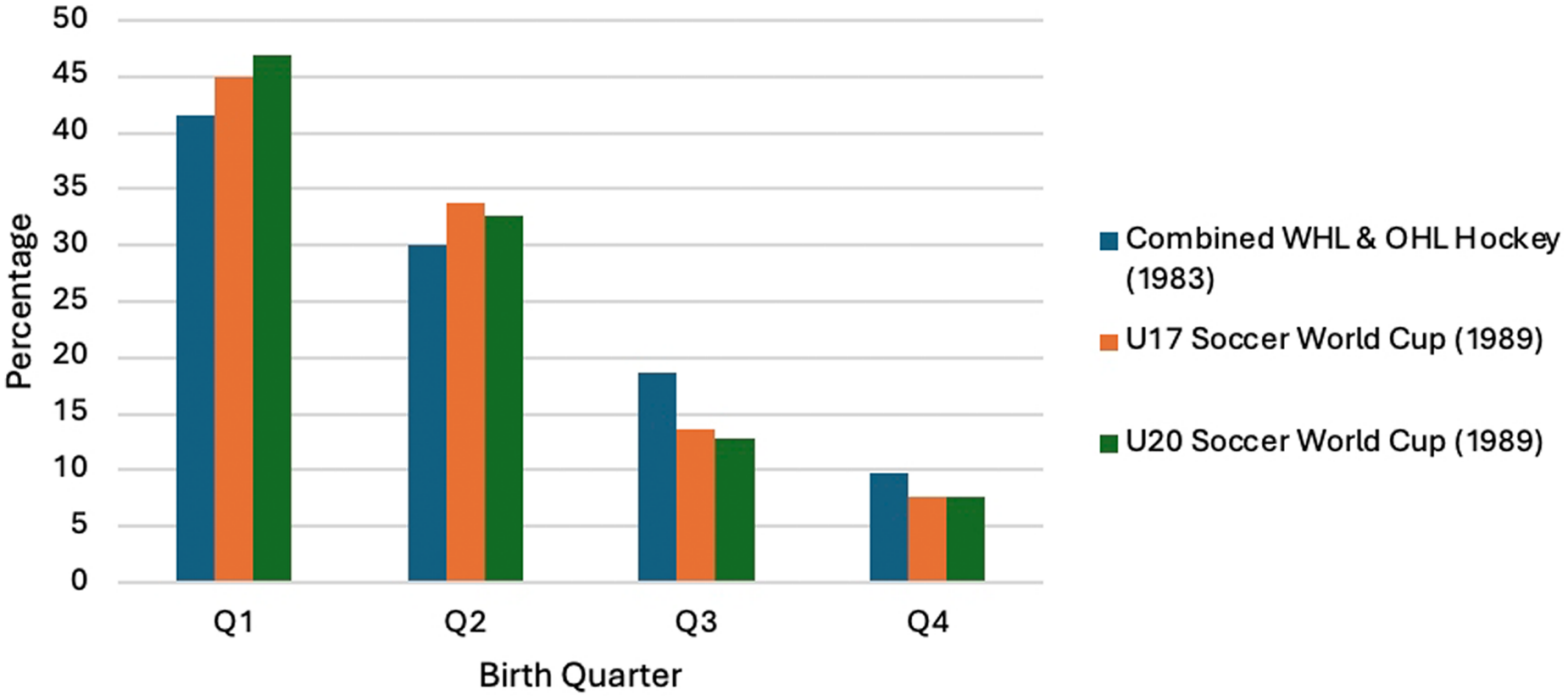
Early RAE studies.

Subsequently, A. H. (Gus) Thompson and I obtained the birthdates of 1,048 players from 1983 rosters of teams in the Western (WHL) and Ontario (OHL) Major Junior A Hockey Leagues. (These were generally considered two of the main developmental leagues for professional teams in the NHL). The combined results from these two leagues were striking. The number and percentage of players born in the four quarters (Q) of the year were: Q1 (January–March) 435, 41.5%; Q2 (April–June) 315, 30.0%; Q3 (July–September) 196, 18.7%; and Q4 (October–December) 102, 9.7%. Remarkably, over four times more players had been born in January, February and March than in October, November and December. These results (2) are found in Figure 1.

Several years later Phil Legault, who at the time was employed by the Canadian Soccer Association, told us that he suspected that RAEs would be strongly evident in soccer (football) world-wide. Working together, the birthdates of players in (1) the 1989 Under17s World Tournament and, (2) the 1989 Under-20s World Tournament were analyzed. The results, which can be seen in Figure 1, were published in “Family planning: football style: The relative age effect in football” (6).

This paper demonstrated striking RAEs from both World Tournaments even though each tournament was comprised of 16 different nations. In fact, the distribution of birthdates was strongly, if not identically, parallel to the findings of the Canadian Major Junior A Hockey Leagues. Again, the cohort analysis by “Quarter” indicated that: over 40% of the players were born in Q1; over 30% in Q2; approximately 13% in Q3; and less than 8% in Q4. Basically, the results indicated that soccer players born in the Q1 months, were five times more likely to be selected to play International Youth Elite Soccer than players born in Q4.

## The development of the relative age effect

Clearly, these findings demonstrated that in hockey and soccer, success was significantly impacted by the inequitable and unfair opportunities created by a player's birthdate. To understand the development of RAEs in hockey, we decided to analyze the birthdates of all players in a large minor hockey league association. To this end, the Edmonton Minor Hockey Association (EMHA) provided team rosters and birthdays of all 7,313 players registered for the 1983–84 hockey season.

The EMHA was organized into “age grouped” divisions and “competitive-based tiers” or leagues. The divisions (under 8; 9 & 10; through to 19 & 20) were comprised of those players born between January 1 of the first year to December 31 of the following year. Each division included 2, 3, or 4 competitive-based leagues.

Players within each age division were annually assigned to a particular team and league based upon skill performance and assessment by the leagues coaches. First, players were chosen for the highest tier teams; followed by selections for the second level teams and so on until all players were placed on a team in an appropriate performance-based league. Basically, the lower tier leagues were recreational, whereas the higher tiers were progressively more competitive with the top tier teams being highly competitive “Rep” (representative) teams for elite players.

The rosters of all EMHA teams were examined in relation to the birthdates of the players and the results were reported in “Birthdate and Success in Minor Hockey: The Key to the NHL”, (7). From this article three basic principles can be identified that produced the “Relative Age Effect”.

### Performance-based selection

Players are assigned to a particular league/tier based on the assessment of their hockey skill or performance. As the older players in an age group will generally be more experienced, physically mature, bigger and stronger, it is not surprising that the top tier teams predominantly include the relatively older players. The makeup of the top tier teams in the EMHA clearly demonstrated this relative age effect.

### Differentiated experience

The purpose of the leagues within each division is to facilitate equitable participation and a levelling of competition for all players. However, because of the skill and performance differences between the tiers, different programs and experiences are created to meet the needs of the players. For example, competition, practice time, number of games, quality of coaches, equipment, etc. are generally varied with the highest tier players gaining many advantages. Over time, these differentiated experiences further exacerbate the relative age differences that were created by the selection procedures.

### Participation rate

The RAE was not evident in the “under 8 through 10 years” division probably because in the beginning all boys wanted to play hockey. However, starting with the 11-year-olds, lower participation numbers of the younger players contributed to the RAE. Presumably, younger players in the age cohort had dropped out for lack of success and enjoyment.

To summarize, it appears that the following conditions during childhood will lead to RAEs: (1) Children are grouped in age cohorts to facilitate the organization and delivery of an activity; (2) Children are selected and assigned to differentiated groups in the their age cohort based upon such factors as size, skill, or achievement; (3) These differentiated groups usually experience different program opportunities; and (4) Children who are disadvantaged tend to “drop out” and stop being participants in the activity.

### Hypothetical examples

To depict RAEs with an example, consider the following two hypothetical player experiences in minor hockey. Both Sam and Mike were enthusiastic hockey fans and with their parents' support began playing minor hockey when they were eight years old.

Sam was born on February 9 and as the minor hockey league was structured with age groups based on January 1 to December 31, Sam was one of the older and more mature boys. At the organizational practice, where the players demonstrated such skills as skating and stick handling, Sam stood out from most of the other boys because of his size, speed and coordination. Sam was one of first players “drafted” and found himself on a team in the top tier. As a “top tier” player, Sam had more practice time, played more games and had more chance to travel and play in tournaments. Each succeeding year, Sam was one of the first boys chosen and he continued to improve and succeed. As a young teenager, Sam, his parents and his coaches, believed that he could realistically receive a scholarship to play university hockey or be chosen to play Major Junior hockey.

Mike, who turned out to be a late maturer, was born on November 15. In his first few years of hockey, Mike enjoyed the game, and his parents appreciated that despite his smaller size, he received equal playing time. However, Mike and his dad had hoped that he would get more time to practice and an opportunity to play in some tournaments, but that seemed to be reserved for the higher tiered teams. And, by the time he was 12, Mike had lost interest and motivation for recreational hockey. As weekend practices were interfering with family ski trips, and because Mike wasn't experiencing success, he dropped out of minor hockey. Later, as an adult, Mike started going to the rink again and found out how much he missed the game. Sunday morning hockey scrimmages with his buddies became the highlight of his week. Mike often wondered how his hockey experience would have been different if he had been born two months later on January 15.

## Hockey and soccer after 40 years

The imaginary stories of Sam and Mike underscore the unfairness of the minor hockey system in 1984. And the occurrence of similar RAEs in International Youth Elite soccer, certainly highlight the need for change. One wonders how players, coaches, parents and communities in general, can accept and continue with systems that are so biased and unfair that children born in the last quarter of an age cohort are up to five times less likely to succeed than those players who were in the older age cohort.

**Figure 2:**
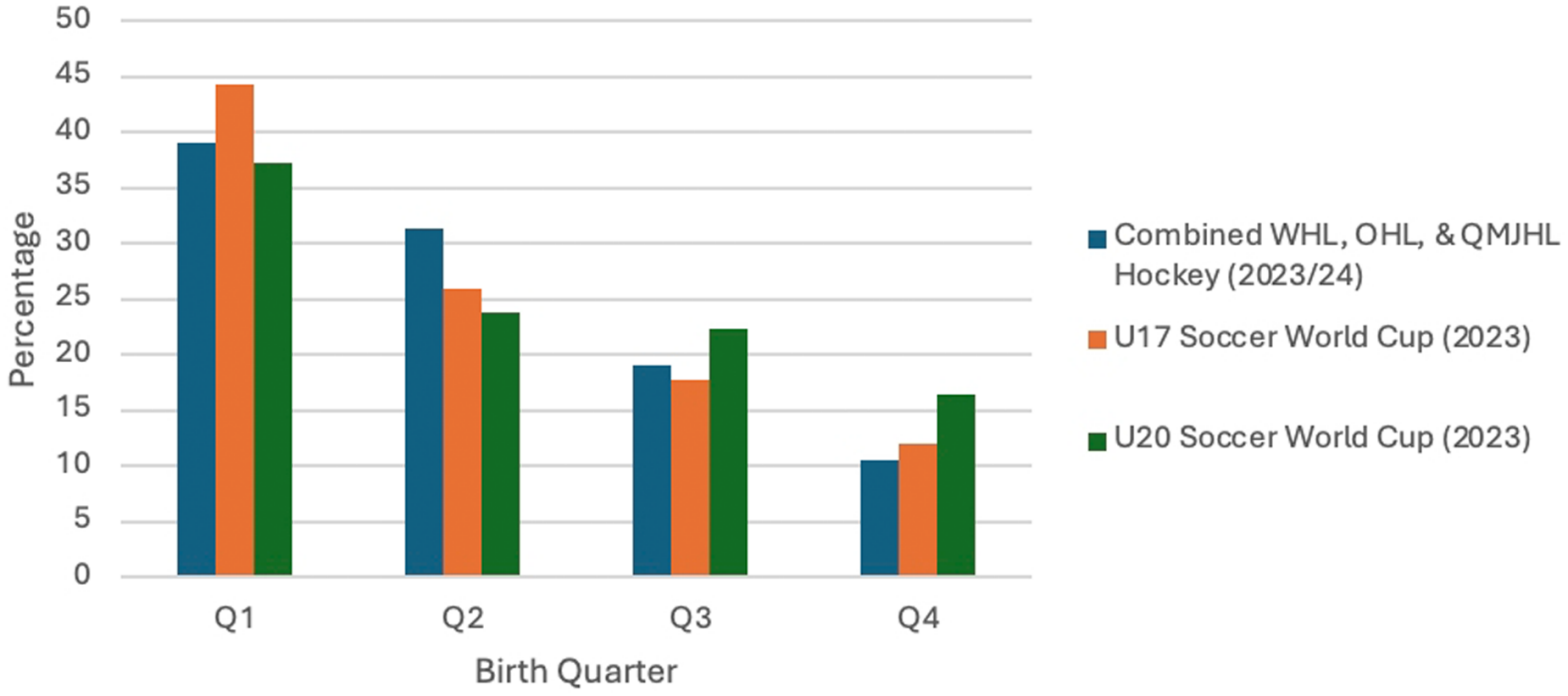
Replicating the early studies of hockey and soccer.

Given these striking differences and the disadvantages for the younger players, it would seem reasonable to expect that after 40 years, knowledge would have been gained and actions taken that would mitigate, or at least substantially reduce the size of RAEs. To consider this expectation, an informal analysis using data from 2023 was carried out for each sport. The results are found in Figure 2. Clearly, simple visual and descriptive comparisons between the hockey players in 1983 (see Figure 1) and those in 2023 indicate that RAEs are essentially identical. (It should be noted that the hockey data has been expanded through addition of players from the Quebec Major Junior Hockey League.)

Regarding the soccer data, there are two notable differences between the 1989 and 2023 tournaments. First, 2023 tournaments comprised 24 national teams rather than the 16 represented in 1989. Secondly, and perhaps more importantly, the age cohort for the 2023 teams had changed to January 1 to December 31; whereas, in 1989, the age cohort was established between August 1 and July 31. Regardless of these two changes, the soccer data also strongly suggest that very little has changed.

The consistency of the data from players and leagues 40 years apart is remarkable. It is apparent that for males aspiring to play professional hockey, those born between January and the end of June are still more than twice as likely to have success than those born from July to December. Or, it can also be said that those born between October and December are four times less likely to be successful than those born in January, February, or March. And, for those males who desire to play elite youth soccer on their journey to a professional career, their birth month provides the same substantial advantage or disadvantage that defines success in hockey. Obviously, RAEs selectively privilege individuals born early in the cohort year with unearned advantages. While at the same time, those born late in the cohort year encounter disadvantageous conditions that have a high probability of inhibiting their success.

## Other areas impacted by RAEs

This chapter has been focused largely on hockey and soccer research. This choice was made to reflect on our early studies and to update them in the context of the 40th anniversary of the “Relative Age Effect”. Of course, recognition and praise are clearly due to the large volume of RAE research that has taken place over the years. Investigating the role of RAEs in a wide variety of sports (8) continues to capture the interest of most researchers.^3^

It is beyond the scope of this paper, but the role of RAEs in education and associated areas should be briefly reviewed. I have found it both interesting and informative to consider that the RAEs in hockey and soccer provide a useful metaphor or, model for understanding the effect of RAEs in education. Comparatively, K-12 education and youth hockey are quite similar as schools are also generally organized into grades based upon age cohorts. And these age cohorts renew annually and continue for upwards of twelve years.

Research in education and associated areas has unsurprisingly demonstrated that RAEs develop because of procedures similar to hockey and soccer. In addition to age cohort grouping, K-12 education often includes specialized program enrolment and opportunities based upon RAE performance and achievement. Interestingly, the reduced participation rate for disadvantaged young children generally observed in sports activities is not found in schools as attendance is mandatory. However, RAEs have been identified in significant areas of education. For example, RAEs have been found in (1) University participation rates (10–12); (2) Academic achievement (13, 14); and (3) Special education classifications such as in Gifted and Talented programs and children who are Learning Disabled (15–17).

Further, a strong case can be made that some areas of personality and mental health (18) emerge as corollaries from the effects of the child's position in the school age cohort. For example, RAE research has demonstrated that leadership abilities (19, 20) are related to the advantages enjoyed by the older students in the school-based age cohort. Levels of self-esteem (21) are generally related to RAEs, and unfortunately, younger students in the school-based age cohort have been found years later to be more prone to suicidal behavior than their older classmates (22). It is interesting to hypothesize that the negative mental health and personality outcomes related to the younger members of the school-based age cohort might emerge as a behavioral accommodation to mandatory school attendance and inflexible age grouping.

## Closing thoughts

The past 40 years have produced a wealth of RAE research. One indicator is that our first article (2) has received almost 700 citations and the soccer article (6) has been cited over 400 times (Google Scholar). Although RAEs have now been identified in many different sports and educational areas, it appears that research addressing factors or procedures to reduce RAEs have been minimal. After 40 years one would have thought, or at least hoped, that procedures for mitigating RAEs would have been found or were being investigated.

Unfortunately, it seems that few people are aware or bothered by the unfairness of RAEs, and to my knowledge formal surveys of RAE awareness have not been done. Over the years, discussions that I have initiated with a range of people suggest widespread unfamiliarity. And for those who are aware of RAEs, discussions often lead to comments such as “It's just swimming, or hockey, or football”, or “There are enough professional players”, or “Why should I care?”, or my cynical favorite “Life is not fair”. Indeed, these attitudes can be found in all categories of stakeholders: athletes, parents, coaches, educators, policy makers and politicians. Clearly, those of us interested in mitigating factors must provide leadership to create awareness and concern for the negative impacts of RAEs.

Nevertheless, changes that could minimize RAEs have been proposed (23); and, reviewed (24). Suggested solutions have included: (1) Changing the size of the age cohort group; (2) Alternating the cutoff dates of the age cohort; (3) Reducing competition with additional focus on skill training and development; (4) Delaying the age for performance-based selection; and other options. However, such suggestions cannot be implemented without evidence of effectiveness and, realistically it is very difficult for researchers to gain permission and consent to try such innovations. Without evidence, there is no motivation or, imperative for policy makers to revise programs that are long-standing, traditional, and expensive to change.

Of course, the fundamental ingredient in the search for protocols, activities, structures, or systems that would minimize or eliminate the RAEs in sports, depends on research. It is re-assuring that RAE researchers are increasingly focusing on these issues. For example, research investigating effectiveness of age-ordered shirt numbering [(25) and (personal communication, Dixon and Horton, 2024)] is encouraging. And a recent publication (26) demonstrated that changing the comparative selection procedures for swimmers, some top talent athletes who previously would have been rejected were now retained. Stephen Cobley (personal communication, 2024) recently shared that an Australian swimmer who won a gold medal at the Paris Olympics would have been eliminated by the old selection procedure because of RAEs. Clearly, the success of such research projects should provide the incentive, enthusiasm and confidence for other research groups to accept the challenge of finding much needed solutions to the negative impacts of RAEs in sports.

I believe that it is reasonable to expect that RAE stakeholders and the relevant organizations should be aware and informed of RAEs and their negative consequences. The past 40 years has produced volumes of RAE information and enthusiastic researchers have reliably reported the results in peer reviewed, respected journals. Unfortunately, the stakeholders who need this information do not usually read professional journals. It is “much easier said than done” but research findings need to be communicated broadly and, in a style and format that is accessible to the various audiences of RAE stakeholders, including journalists, and readers of popular publications. This observation is probably relevant to all areas of human research; however, few other areas so directly impact, both positively and negatively, the life choices and successes of most people.

To conclude, the presence and scope of RAEs in sports and education is both remarkable and wrong. Clearly the randomness of a person's birthdate should not be related to one's success and overall achievement in life. The unequitable and enduring effects of RAEs make a compelling case that as researchers, we cannot and should not remain silent, neutral, or restrained. Every time and everywhere that RAEs are evident, there are individuals who are privileged and gain an advantage; and, a comparable number of people who are disadvantaged, limited, or excluded. These outcomes are unfair, unjustified and wrong. After 40 years, the time to address these issues is long overdue!

## Data Availability

The original contributions presented in the study are included in the article/Supplementary Material, further inquiries can be directed to the corresponding author.

## Footnotes

^1^It is recommended that readers listen to “Outliers, Revisited”, Season 7 of the podcast “Revisionist History”

^2^As I was concluding the preparation of this manuscript, I became aware of Simon Grondin's publication “To be or not to be born at the right time: lessons from ice hockey” (4). In this paper Simon contemplates his early RAE publications; comments on the present state of RAE research; and recommends that more attention should be paid to mitigating RAEs. This paper explores a number of issues discussed by Grondin, and therefore, it should not be surprising that in our “40th anniversary” reflections, we are generally agreed on the path forward.

^3^Parenthetically, this retrospective has largely dealt with male-based studies. For a discussion of RAEs in female populations, readers are referred to “Relative Age Effects in Female Athletes” (9).
